# On Some of the Latent Causes of Insanity
*Leçons Cliniques de Médecine Mentale, par M. Fahet, Paris, 1854.


**Published:** 1854-04-01

**Authors:** 


### THE JOURNAL

OF

### PSYCHOLOGICAL MEDICINE

AND

### MENTAL PATHOLOGY.

APRIL 1, 1854.

### Art. I.—ON SOME OF THE LATENT CAUSES OF INSANITY.*

The entire universe, according to transcendental speculations, is a unity in which all the parts are related and mutually dependent. A remark of Feuchtesleben' may be quoted as an illustration of one department of this mutual dependence: "Could we penetrate into the secret foundations of human events, we should frequently find the misfortunes of one man caused by the intestines of another, whom the former endeavoured to inspire with sympathy in his fate at a moment when the frame of mind of the latter was affected by impeded secretion. An hour later and his fortune would have been made." But his fortune not being made—what then? His future course might be like that of Hamlet—

> " ——— and he, repulsed,
> Fell into sadness, then into a fast,
> Thence to a watch, thence into a weakness,
> Thence to a lightness; and by this declension
> Into the madness wherein now he raves."

And thus through events, in a direct series, from the impeded intestinal action of one man we pursue another to an asylum.

We propose to trace the origin and progress of some of these "sympathies" between mind and matter, and man and man, and investigate their relations to insanity; but the sympathies we shall

* Leçons Cliniques de Médecine Mentale, par M. Fahet, Paris, 1854.

investigate will not be so obvious and potent as the preceding. We shall endeavour to get below the surface, and detect some of those with more hidden springs; or, disentangling the chaos of facts which lie to our hands, try to catch hold of a link "of that electric chain with which we are darkly bound."

In whatever way or from whatever point we view the reciprocal influence of body and mind, and of organisms on organisms, we are finally brought to the physiology and pathology of the nervous system. Analyzing the phenomena as they present themselves in man in relation to that system, we are as inevitably led to the cerebrum as the instrument of the mind, and the medium of communication between it and matter. Struggle as we will, to free us from the Material which clothes the Immaterial, we always find ourselves at the same point, and cannot help but acknowledge how inextricably bound up are all our mental operations with that wonderfully constructed but most perishable apparatus. It is true that, in the pride of intellect, philosophers have despised the teachings of physiology and medicine, or, in the vanity of ignorance, have wholly rejected them; it is equally true, too, that the moralist has vainly denied to the material organism all control over man's volitions; it is equally true that the legislator and the judge have contemned that—to them—maudlin doctrine, which teaches that man is irresponsible for his acts, on the ground of uncontrollable automatic operation of the material instrument, if so be that he knows and is conscious that his acts are evil; it is equally true that the untaught man of action has worked in utter ignorance that he used an instrument at all. But the practical experience of the psychiatrician is, nevertheless, opposed to the conclusion of the philosopher, the moralist, the legislator, the judge, and the untaught man of action. That experience is worth folios of hypotheses, for it teaches us the humiliating truth, how utterly dependent are the highest flights of the imagination, and the deepest conclusions of the intellect, on the working of the material instrument, and on its right relations to external agencies.

> "Man is but man, inconstant still and various;
> There's no to-morrow in him like to-day;
> Perhaps the atoms whirling in his brain
> Make him think honestly this present hour;
> The next a swarm of base ungrateful thoughts
> May mount aloft."

Before particularizing the sympathetic influences we propose to discuss, it will be well to indicate in outline the anatomical and physiological position of their recipient—the cerebrum. All mental phenomena depend upon alterations in the functional activity of the latter; a cessation of this activity, as in profound sleep, is coincident with an abolition of mental action. Now the cerebrum is made up essentially of a series of ganglia, or, in other words, of concentrated and connected masses of gray nervous matter, and it is with the *dynamical* changes which go on in these centres that mental phenomena are coincident. All the sympathetic influences, therefore, which we have to consider, must reach these ganglia, and induce those requisite dynamical changes without which mental phenomena are never manifested.

If these primary considerations be granted, it follows that we must inquire carefully, in the first place, into the laws of action of the cerebral ganglia. This task has, indeed, been often undertaken. Every available means of research has been put in operation; the eye of the microscopist has penetrated, to their ultimate organization and disclosed the caudate vesicle, nucleus, or cell; the scalpel of the anatomist has traced their connexions with each other fibre by fibre; the test-tube and scales of the chemist have demonstrated their bio-chemical composition; the researches of the pathologist have shown how often morbid changes in structure or in chemical composition accompany morbid changes in functional activity; and, we must add, how often they do not. But, with all this admirable industry and skilful inquiry, we as yet know nothing of those intimate changes in the instrument, with which mental operations are coincident. Is it possible we can ever attain to a knowledge of them? The answer must be in the negative. If we know not the nature of the dynamical changes which occur in brute matter, coincidently with chemical, electrical, and magnetic phenomena, how much less can we know those which occur in organized matter? It is clear that we can only in the latter, as in the former, study the dynamical changes through the phenomena.

Vivisections, physiological experiments, and pathological observations, have been rendered available to a large extent in determining the functions of less complicated and less important ganglia than the cerebral; but, as to the latter, they have been of little avail, nay, have led experimental physiologists into errors which the philosophical physiologists have escaped. Thus Dr. M. Hall, finding that by pricking, tearing, irritating with chemical irritants, and otherwise mechanically acting upon the spinal chain of ganglia, and its continuation into the encephalon, as far as the tubercula quadragemina, he could excite the motor machinery into action, he came to the conclusion that the part of the great central axis, so responsive to mechanical irritants, was a distinct portion of it; and he drew a sharp line of demarcation between that tract and the cerebrum beyond, not only as to structure, but also as to function. Yet nothing could be more

N 2

fallacious. In the application of his mechanical irritants to the central ganglia, he could only imitate artificially those stimuli which naturally reach the gray masses and excite their functional activity; he therefore imitated those only which act upon the ganglia through the nerve-fibrils running to them from the skin, mucous surface, and tissues in general—or the nerves of what is termed common sensation, —and that in a very rude and imperfect way. All impressions reaching the central axis from special apparatuses, as those communicated through the senses of taste, smell, hearing, and vision, are, in fact, altogether inimitable by the methods ordinarily adopted by vivisectors. The nearest approach to natural stimulus from these sources is perhaps obtainable by electricity or galvanism; but even with regard to these means it is very doubtful, indeed, whether any such dynamical changes could be induced by them in the cerebral ganglia, as are consequent upon impressions reaching them through the natural channel of communication from without.

Nor is there more hope from pathological research. Many a time and oft has it been alleged that the most careful dissection of the cerebra of the insane has revealed no change whatever in the gray matter; and many a time and oft has the hasty induction been made, that therefore we must not look for the seat of insanity in the encephalon, and that any morbid changes which may have been observed, must be considered rather as the effect than the cause of deranged functions. A careful consideration, however, of the essential nature of insanity, must inevitably lead to the conclusion, that in the cases which come strictly under the term, none other than a dynamical change, *analogous* to that of health, can be expected. A *manifest* structural change in the instrument of mind can only happen coincidently with *abolition* of the mental faculties; whereas, in true insanity, the faculties remain, and are *perverted*. When the functional changes which induce insanity have passed into structural alteration, and the intimate tissue is so compressed by congestion or effusion, or so changed by disintegration, or imperfect nutrition, that it ceases to fulfil its functions, we have *loss* of mind, not *disorder* merely. That this is the common termination of many cases which end fatally, is simply a truism; the knowledge that the disorder of the intellect has so ended in structural change and in abolition, helps us nothing in the comprehension of the changes which occur during insanity itself, for we know nothing of the dynamical relations of the material instrument to the immaterial power.

Is there, then, no method, it may be asked, by which a better knowledge of the condition of the cerebrum in insanity may be obtained, and by which we may especially *learn* the laws of action of those influences which, under the term *sympathies*, have so large a share in the causation and cure of cerebral disorder? We think there is a method available to these ends, and we shall now proceed to trace it out by the way of synthesis and analysis.

The propositions from which we can start with safety, are two, namely, 1, that the cerebrum is the organ of the mind; and, 2, that it is organised matter subject to the laws which regulate organised matter. If we trace its relations through general and comparative anatomy, we arrive at the conclusion, that it is constituted of ganglia, subject to the same laws of action as the ganglia of the spinal cord. This principle is based mainly on two great facts, namely, 1, that the cranium being homologically nothing more than the development of four vertebræ, the contained centres correspond to the central axis contained within the vertebræ, and consequently that, like the spinal ganglia, the cerebral are functionally subject to the laws of automatic, spontaneous, or reflex action. Physiological researches have now almost established this proposition, for the doctrine of "unconscious cerebration," recently propounded by an eminent neurologist, is none other than a development of this doctrine of *reflex cerebral function*. It is true that that lucid thinker connects sensation with the sensory ganglia as a necessary element in their functional activity; but it is obvious that with unconscious reflex action above these, in the hemispherical gray matter, and with unconscious reflex action below them in the spinal gray matter, this apparent exception will ultimately cease to be exceptional, and the great and fertile principle will be adopted, that the instrument of mind itself, like other portions of the nervous system, may act independently of mind, but automatically, adaptively, and as if regulated by conscious mind.

The importance of thus placing the cerebrum dynamically, in the same category with other portions of the nervous system, becomes more obvious, when we find, on tracing that system to its elementary constituents, that we must place it dynamically, in the same category with other living matter. After we have traced the analogue of the spinal cord of the vertebrata through the ganglionic chain of the articulata, we have still to push our analogies lower and lower, until we come to animals with a single ganglion, or multiplication of it, endowed with the simplest attributes in relation to external influences, but nevertheless presenting the same dynamical functions as the highest, inasmuch as within its more limited sphere of action that simple ganglion acts just as automatically, adaptively, and as if regulated by conscious mind. The transition, anatomically, from these lowest forms to those organisms in which no trace of nervous system can be found, is not difficult to follow; but it may appear startling to endow the latter, physiologically, with powers which are usually thought to be the special endowment of nervous tissue. Yet to no other conclusion can we come, if we look at the functional dynamics of the simplest microscopical cell, for in this as in the series of ganglia, from the highest to the lowest, we still find the common principle of action, namely, ultimate adaptive action within the narrow world in which they act, as if regulated by conscious mind. We are thus, therefore, brought by our analysis to the ultimate cell, as the prime type of that system of dynamics which has its highest elaboration and development in the human brain.

How, then, does the cell manifest its adaptive and quasi-reasoning powers? In two ways—First, in evolution and development; secondly, in actual life. The natural history of the infusoria, is a grand illustration of the latter; the embryonic, or primordial cell of the human organism, of the former. To the contemplative mind, nothing is more wonderful amongst all the properties of vitalized matter than the successive series of vital processes, which, commencing in the primordial germ, are continually unfolded, each more complex than its predecessor, each giving rise to more marvellously constructed instruments, and more wisely adapted actions than the other, until the human brain is elaborated with the full perfection of the mature intellect, and the human form developed in all its beauty and glory. The instinctive life of the infusoria is an impressive illustration of what living matter is capable. According to botanists, they are Algæ; yet they are nothing more than simple cells, having their walls strengthened by silica. If these cells, in so low a step of the scale of organism, present such remarkable endowments, why should he attribute less of the adaptiveness and unconscious mind manifested by their acts to the cells of the gray matter of the nervous system in man, and the higher vertebrata? When writers speak and think of the brain as mere matter, they are little aware with what rare and wonderful powers the most insignificant and microscopic cell is endowed.

Cell-life in vegetables is a social life. The cells have a separate existence and division of labour. "The great object which I have kept in view throughout," Mr. Quekett observes,* "has been that of endeavouring to impress on you the fact, that each cell of a plant should be considered as having an independent or individual existence; that in one situation it may secrete colouring matter, in another, starch, gum, sugar, oil, &c., and in another the material for the reproduction of its species." Thus, certain cells, or classes of cells, have functions assigned to them, in the scheme of vegetable life. In the

* Lectures on Histology, p. 113.

scheme of animal life, the special organ of adaptation to definite ends —the gray ganglionic matter—is divided into parts having special functions assigned to each; their laws of action being writ (to use the words of Prochaska) in the nervous pulp, or, in other words, the cells of the ganglia have special functions assigned to them.

Now it is in the greater multiplicity of these special adaptations, that the higher organisms excel the lower; it is therefore in this exceeding multiplicity of functions that the human cerebrum excels all other cerebra. But every individual is ONE—a unity. Hence the necessity of intimate union, and of common action in these specialities. They all require to be *co-ordinated*. In the co-ordination of all the dynamical processes to a common end—namely, the well-being of the whole as a *unit*—we have that view of the processes known as *Life*, and those mutual influences developed termed *sympathies*. "Thus, turning to what is physiologically classified as the *vegetative system*, we see that the stomach, lungs, heart, liver, skin, and the rest, must work in concert." We quote from a very able essay in the Westminster Review.*

"If one of them does too much or too little—that is, if the co-ordination be imperfect, the life is disturbed; and if one of them ceases to act—that is, if the co-ordination be destroyed, the life is destroyed. So, likewise, is it with the *animal* system. Its component parts, the limbs, juices, and instruments of attack and defence, must perform their several offices in proper sequence; and farther, must conjointly minister to the periodic demands of the viscera, that these may, in turn, supply blood."

It is the nervous system which is the grand medium of communication between every part of the organism. The function of that system is, therefore, emphatically *internuncial*, as John Hunter most happily termed it. But the nervous system is also the great co-ordinating apparatus. It is, therefore, more especially the seat and source of all those adaptations and combinations of machinery by which the organism is maintained in being, preserved, and reproduced. In the motor system, we see how strength thus results from the co-ordination of action; "for it is produced by the simultaneous contraction of many muscles and many fibres of each muscle; and the strength is great in proportion to the number of these acting together—that is, in proportion to the co-ordination. Swiftness, also, depending partly on strength, but requiring also the rapid alternation of movements, equally comes under the expression. So, too, is it with agility; the power of a chamois to spring from crag to crag implies accurate co-ordination in the movements of many different muscles, and a due subordination of them to

* The Westminster Review, New Series, vol. i. (April, 1852) p. 472.

the perceptions. The definition similarly includes *Instinct,* which consists in the uniform succession of certain actions, or series of actions, after certain sensations, or groups of sensations; and that which surprises us in instinct is the accuracy with which these compound actions respond to these compound sensations—that is, the *completeness* of their co-ordination. Thus, likewise, it is with *Intelligence,* even in its highest manifestations. That which we call *rationality* is the power to combine or co-ordinate a great number and a great variety of complex actions for the achievement of a desired result."

Now, if from extraneous causes, the co-ordinating apparatus be so altered that this uniform succession of certain actions, or series of actions, with their corresponding sensations, or groups of sensations, be interrupted, and a link in the chain be pushed out of its proper position or severed, *disorder* will take the place of order, and there will be abolition, perversion, or irregularity of the entire series of processes. If, then, this condition arises in the co-ordinating apparatus of the intellect, of the feelings, of the emotions, of the appetites, of the instincts, we have various manifestations of DISORDER *of the mind.* If, on the other hand, it take place in the co-ordinating apparatus of the muscular system, we have the various manifestations of *disorder* in the motor portion of the nervous system, grouped under the term MOTOR *neuroses.*

It is thus, therefore, of primary importance in the investigation and cure of disorders of the mind, to trace the morbid phenomena up to the broken or strained link in the series, and determine accurately the exact relation of the breach in the continuity of the series to the immediate antecedents, or the *cause.* That cause is often none other than that influence of one series upon another which is termed *sympathy;* and we shall now proceed to illustrate by practical applications the method of investigation to be followed in determining the origin, nature, and relation of these sympathies, in reference to the etiology and treatment of insanity.

*Blood Sympathies.*—The lowest organic process is growth; in the cell it involves two processes—accretion and disintegration, nutrition and removal, repair and waste. This process is therefore the essence of life; so soon as the one ceases the other is extinct. The entire co-ordinating apparatus of the organism will therefore be directed to three great ends—namely, 1, supply of material; 2, supply of oxygen; 3, combination of material and oxygen in nutrition. Now the blood is the medium by which these three processes are perfected, and is the great agent, therefore, by which the co-ordinating processes are put in operation. Hence it is that the blood acts so incessantly in the co-ordinating apparatus—the nervous system. But if the blood be so changed in its composition that it develops violent or irregular action in the nervous system, what will result? Let us examine a little more closely.

We will suppose that the supply of nutrient material has been wholly suspended for a certain period, or, in other words, that hunger has been experienced. If nutrient material be supplied, and the appetite appeased, the chain of sympathies is completed, and the end is gained. But if food be not supplied, in a while disorder begins. The entire machines of the organism are co-ordinated to one great end, and are gradually involved in the sole business of seeking food, to the neglect of their proper functions. The actions become impulsive and instinctive; the higher powers are in abeyance, or are perverted; and the man becomes a mere animal. He becomes this the more emphatically in proportion as the restraining power of the latter over the appetites and instincts was originally small, and *vice versâ*. Histories of shipwrecks and marches illustrate this state of things. It is the same when an insufficient supply of water is sent to the blood, and *thirst* is experienced. Major Mitchell made three expeditions into the interior of Australia, taking with him "convicts on leave." His party was sometimes exposed to extreme privation, especially of water, and he remarked that it was the worst characters that had the least control over their appetites under these circumstances. "It was a standing order," he says, "which I insisted upon being observed, that no man should quit the line of march to drink, without my permission. There was one, notwithstanding, who never could, in cases of extremity, resist the temptation of water, and *who would rush to it, regardless of consequences.* Now this man continued to be an irreclaimable character, and in six years after had lost all the advantages he gained by his services on this occasion."*

We are inclined to think that this general blood-sympathy will throw light on the origin, nature, and treatment, of those cases of mania which commence with, or during their course exhibit, absolute anorexia. In the treatment of ordinary cases of mania at home, such is the terror and disgust inspired, and such is the carelessness and ignorance displayed, that the patient is not supplied with necessary food and drink. The poor sufferer is himself unable, perhaps, to indicate his desires and wants, or it may be that the condition of that portion of the sensorium in which the sensations are felt, is not capable of transmitting the impressions to the consciousness, although it can draw the rest of the co-ordinating apparatus into the chain of morbid actions; and so it happens, that because food and drink are not asked

* Three Expeditions into the Interior of Australia, &c. By Major Mitchell. Second Edition, vol. i. p. 58.

for, they are not given. Often have we been struck with the weak, hoarse voice, the clammy tongue, cleaving to the mouth, the haggard look of the poor maniac under these circumstances; often, too, by touching their hidden sympathy, we have been able to re-construct for a moment, the broken chain of co-ordinations, and so far awake the sufferer to ordinary consciousness, that he has greedily gulped down the cooling draught of water presented to him. In many cases, indeed, of this kind, the sole remedies are kindness, food, and drink.

Certain articles of diet are necessary to the proper action of the nutrient apparatus. If these be wanting in the blood, the desire arises for them. Hence spring what are termed *longings*; hence, also, *depraved appetite* for food. Fresh vegetable food is one of these. The case of a furious maniac came under our notice, whose history well illustrates the value of attending to the philosophy of this kind of blood-sympathy. For many weeks he was destructively maniacal, partly against persons, but only from sudden bursts of irritation; principally against things, so that not a chair or a window escaped his violence. He broke 200 squares of glass in a very short time. What was remarkable in his case was this, that he ate ravenously of his food all the time, but however well-fed he would not let a blade of grass, or a weed, or a green thing grow in the airing-court in which he walked. If his hands were restrained, he knelt down and tore up the weeds with his teeth. Noting this instinctive appetite, we directed that he should be supplied with uncooked carrots, celery, &c., *ad libitum*, believing that it was an indication of a morbid condition of the blood,—probably an exciting cause of this maniacal state. The result justified our deduction; and as hopeless a case as it has been our lot to witness, was perfectly restored to health and sanity. Other illustrations of these modifications of the natural appetite from blood-sympathies, are presented in cases of *chlorosis* in young females, of pregnancy in others, and by negroes labouring under that kind of appetite which leads them to eat earth, and in which there is obviously a condition of the blood almost identical with that of chlorosis. Ben Jonson, in his play of the "Magnetic Lady," gives a summary of the *desideranda* in cases of this kind:—

——— "She can cranch
A sack of small coal, eat your lime and hair,
Soap, ashes, loam, and has a dainty spice
Of the green sickness."*

*Bulimia*, *Polydipsia*, and *Pica* in all its varieties, may therefore be excited by a morbid condition of the blood awaking abnormal actions in the co-ordinating apparatus. Perhaps, also, to this class of phenomena

* Act i., scene i.

may be referred that still more lamentable form of depraved appetite, well-known to the psychological physician as *oinomania*, the most characteristic symptom of which is an irresistible craving for nervine stimuli, but more especially the alcoholic.

These results of morbid blood-sympathies are comparatively normal if we take into consideration another group, namely, those characterized by an appetite for what is horrible and disgusting. Some insane persons eat excrement greedily, others any garbage. *Lycanthropia* belongs to this class of depraved appetites—consisting essentially in an appetite for raw flesh. However these may originate—that is to say, whether from disordered action in the nervous system, arising idiopathically, or whether excited by blood diseases, or whether induced by the sympathies of certain viscera (to be presently described), there is this characteristic,—that the morbid appetites developed in these forms of insanity are manifested in lower animals as their *normal* appetites. This is a most important characteristic, and is, indeed, common to numerous aberrations of the instincts and emotions in the human being. How the latter originate is, indeed, a mystery; but they show this much, that there are, in the depths of man's nature, hid away and covered over, if the expression may be used, natural sources of mental phenomena, which are the proper characteristics of brutes. It is as if, in man's mental frame, as in his physical, there may be a retrograde manifestation of organs and uses; as if the brute instincts may appear in him, according to the same law that there is sometimes found a divided lip, or a two-horned uterus—monstrosities of mental development, analogous to the corporeal. The manifestation in idiots of these lower series of co-ordinate acts, is by no means unusual. An idiot girl is delivered, when alone, of a child, and like the females of lower animals, she tears the umbilical cord with her teeth. Dr. Corsellis mentions in his Report for 1851, of the West York Lunatic Asylum, at Wakefield, the admission of a congenital idiot boy, aged 12, said to have been left in a cottage by some gipsies. He is thus described:—

"He is unable to speak, and in appearance and habits partakes more of the brute than the human species, expressing pleasure or disapprobation by a wild cry, or by flapping his arms to and fro like the wings of a bird, and being destitute even of such intelligence as would enable him to be destructive or mischievous. A peculiarity marking the case of this singular child is, that he ruminates his food. When eating, his food is bolted or rapidly swallowed, without mastication. As soon as the meal is finished, the ruminating process commences. A portion of food is raised from the stomach, sometimes by a visible effort, but not always accompanied by eructation; the morsel is then deliberately chewed and re-swallowed. Afterwards, a fresh portion is raised in a similar manner, and the process continues for a quarter of an hour or longer. During rumination, he remains quiet and completely absorbed in the act. If the morsel brought from the stomach is large, he divides it into two portions, retaining one in the fingers, until the other has been masticated and re-swallowed. The regurgitated food has an acid reaction.''

Several points of retrogression are illustrated here; perhaps the instinctive motions indicating pleasure and pain, and the development of the ruminant instinct—a thing necessary, probably, for the nutrition of this idiot, as he would be liable to be neglected in feeding—are the most interesting.

*The Visceral Sympathies.*—There are two groups of the viscera, if they be classed with reference to their relations to the co-ordinating apparatus. We place together in the one the organs which receive and commingle the oxydizable and oxydizing matter—the blood; the organs which circulate it; the organs which depurate it. These are, respectively, the lungs, stomach, spleen, and small intestines; the heart and vascular system; the liver, kidneys, large intestine, and skin. Their sympathies all concern the individual. The organs of reproduction of the species constitute another group; their sympathies are a class to themselves; they all concentrate on the union of the two sexes, and on the offspring resulting from that union. In short, they do not concern the *individual.*

In considering these extensive sympathies, psychologists have hardly discriminated between those which are purely dynamical, those which are functional, and those which are structural or organic. Thus, the uterus and ovaria act upon the stomach, mammæ, kidneys, or intestines, and induce various important functional changes as they act through the spinal cord by a direct physical influence on that centre of impressions and actions. It is not so when the ovaria act upon the encephalon, and bring various reproductive and parental instincts into operation; for the new world of thought and feeling thus opened out seems to depend upon a different chain of causation. So it is also with the viscera; since they appear to have a similar double action on the cerebrum. Let us examine these more in detail.

The *sympathies of the heart and lungs* are two-fold. First, they co-ordinate the internuncial apparatus for the development of the conservative operations of the organisms, in so far as they are directed towards the due oxygenation and circulation of the blood. Any attempt to stop the ingress of air to the lungs, is resisted with the concentrated energies of the whole system; any arrest of the flow of blood through the heart excites unutterable distress. This profound implication of the instinct for existence, and of the instinctive feeling of horror for its cessation, is more or less developed to the consciousness whenever the functions of these viscera are interrupted by structural or functional disease. Hence the indescribable anguish and restlessness experienced in certain diseases of the heart and lungs; and hence the connexion between the latter and hypochondria. The morbid condition of the heart need not necessarily be *structural;* any change in the *innervation* of that organ, sufficient to excite morbid sensations, will act upon the co-ordinating apparatus, and excite the instinct into action. Perhaps in cases of mania, melancholia, and hypochondriasis connected with structural and functional derangement of the heart, the series of consequences and antecedents is probably something of this kind:—Enfeebling influences, of a mental origin, act upon the nervous system and through it upon the heart; its innervation thus becoming deranged it reacts upon the nervous system, which in its turn reacts upon it; and so by alternating influences various cerebral and cardiac diseases are induced. Excessive study, exhaustive amatory indulgence; strong emotions, especially those that are painful, as anxiety, grief, terror, fear, and the like, are well-known to have a direct influence on the heart and lungs. It is now also becoming more certain that, contrarily, the doctrine first advanced by Nasse in 1817 is well-founded, and that the heart has a most important influence on the cerebrum in exciting functional disorder therein.

The remarks applicable to the heart and lungs are applicable to the entire group of viscera, in which the sympathies concern the welfare of the individual; for in the diseases of all it is the instinctive love of life and fear of injury and death which, in the first instance at least, is morbidly developed. From this, as from a common root or stem, spring certain forms of monomania and certain monomaniacal illusions and delusions.

A hypochondriacal patient may remain all his life with no further mental disorder than the groundless anxiety for his health which devours him, and the morbid attention to one or other of the organs in which he feels uneasiness, that characterizes the affection. But he may easily pass into other stages. The instinct itself may be absolutely *perverted*, just as we have seen the appetites are perverted, and then a *suicidal* impulse is developed. Or the disorder may extend from the more simple instinct of conservation, acting irrespective of external agencies to the instinct of self-defence, of which the idea of something injurious to the organism acting upon it from without is the basis. This will again present modifications,—e. g.; the sufferer may *suspect* that his ailments are induced by poison, or by other secret arts, or by mysterious agents, as electricity, witchcraft, diabolical agency, or the anger of the Deity. He may connect some individual with this notion, and the instinct may then become *homicidal;* he may simply feel instinctive horror for his position, and groan helplessly in profound *melancholia.* He may refuse incessantly the food offered him, fearing to be poisoned, or watch the live-long night against his imaginary enemies. Constant anxiety, anorexia, and sleeplessness do their work, and at last the entire intellect gives way, and complete mania is established. Now we do not intend by any means to insist that in no case do these symptoms spring from idiopathic cerebral disease; on the contrary, we think it certain that instances of that kind are constantly met with in practice: all that we urge here is this,—that morbid innervation and disordered functions of the heart, lungs, and other viscera, have a *dynamical* effect on the instinct of self-preservation; that a play of affinities between the cerebrum and viscera is thereby established; that from the morbid development of this instinct other charges in subordinate instincts radiate as from a common centre, and that finally the whole of the co-ordinating apparatus is involved in the chain of morbid causation. In many instances, morbid innervation may predominate; in many there may be simply disordered functions; and it will be right to discriminate in practice as to the two.

*Morbid functional sympathies* arising from the viscera all act by quickly changing the composition of the blood. It is in this they specially differ from morbid innervation, which acts directly on the cerebrum. In disease of the lungs, there is imperfect oxygenation of the blood; in diseases of the liver, kidneys, and large intestines there is imperfect depuration. As to the connexion between cerebral disease of every kind and *renal* disorder, no experienced practitioner can have any doubt: it is even more obvious than the influence of disordered *hepatic* function. Popular opinion certainly accords with pathological research as to the influence of *functional* derangement of the liver in the mental characteristics.

> " * * * * * * * Væ meum
> Fervens difficili bile tumet jecur,
> Tunc nec mens mihi nec color
> Certâ sede manet."
>
> HOR. *Carm.*

There is much difference of opinion as to the extent and nature of the connexion between disease of the *colon* and insanity. Esquirol first distinctly observed that displacement of the transverse colon such that it assumed a vertical position descending perpendicularly into the pelvis behind the os pubis, was a noticeable feature in the pathological anatomy of the insane; he found it in 33 out of 168 bodies of individuals labouring under melancholia. Bergmann published the dissections of 13 cases, in which very considerable contractions were found in the colon.* In some it was likewise displaced nearly in the manner described by Esquirol. In combination with this state of the colon, (we quote Dr. Prichard's summary,) Bergmann found the following morbid phenomena:—plethora of the abdomen and the encephalon; hæmorrhoidal disease; tumefaction of the spleen, liver, and uterus; distention of vessels in the brain. The mental phenomena in such instances are chimerical ideas. The patient thinks he has animals in his entrails, as frogs or serpents: perhaps to this class of cases belong those in which the patient has a conviction that he is without stomach or bowels at all. Guislain confirmed these researches, and attempted to account for the anatomical phenomena by attributing them to inflammation. The question for us to consider, however, is, what relation do they bear to mental disorder? Now on this point it has been forgotten that the colon is a depurating viscus, and that there is an undoubted connexion between the presence of large quantities of offensive fæcal gases and insanity, so that the disease may act on the cerebrum by preventing effective depuration of the blood.

*Direct visceral sympathies* do, however, constitute a large and important group, and to this the disease of the colon just noted may belong. Nothing is more common than to connect gastro-intestinal and hepatic irritation with mental derangement. The right theory, or such a theory as modern neurology supplies, is hardly comprehended. Close observers of diseases of the nervous system, cannot fail to have seen how frequently paroxysmal diseases, sleeplessness, mental irritation, and mania are connected with obscure irritation of the gastro-intestinal mucous membrane. Epilepsy and mania have been traced to intestinal entozoa; there is no more common cause of sleeplessness, than irritation of the gastro-mucous membrane, by an excess of acid in the fluids; and indeed, no better anodyne than a tumbler of cold water to dilute the acid, or an alkali to neutralize it. We have seen sound sleep come on in a few minutes, after hours of restlessness, as if induced by magic by this simple but potent remedy, much more potent, indeed, than any narcotic. In like manner, the copious evacuations of offensive accumulations in the intestinal canal, has been followed by the happiest results. So also the ablution of a hot and irritable skin, or a soothing application to an eruptive disorder, has induced repose of the stimulated cerebrum, when other and apparently more suitable means have failed. They who have witnessed the results of simple means like these, judiciously applied, can readily understand how easily the homœopathic physician will persuade his patient that the infinitesimal dose was the medicinal agent, and not the vehicle or the adjuvants. Chronic disease of these surfaces is no unfrequent exciting cause of mental disorder.

* Prichard's Treatise on Insanity, p. 231.

The *dynamical sympathies of the reproductive organs* constitute a group of singular interest and importance in every way. They are separated by a distinct characteristic from the merely visceral, and thus the study of them is much facilitated, for while the latter involve the instinct of self-preservation, the sympathies of the reproductive organs override it, and co-ordinate the internuncial apparatus to quite different ends. They can be traced also, without difficulty, directly to the influence of the reproductive organs; for these are subject to periodic activity and repose, or can be wholly removed by vivisections or otherwise altered. For the same reasons, observation and experiment can be brought to bear upon a definition of the precise degree and extent to which the organs themselves react upon the nervous system, and enable us to determine exactly what instincts, emotions, and intellectual operations are subject to their sympathetic influence.

We do not propose to go over the well-beaten ground of the psychology of physical love; our task is a much less ambitious one, but not less important. What is wanted, is, to fix the connexion between these organic sympathies and certain forms of mental derangement. Now they appear differently in the two sexes. In the female more obviously, more variously, than in the male. It is in the lower animals, in which there is a periodical *nisus* and repose, that we can best determine the influence of those sympathies, and we accordingly find that, in the entire scheme of animated creation, the *instinct to fight* and use the natural weapons of offence and defence, (supplied to him almost exclusively,) is developed in the *male* animal by functional activity of the reproductive system. Fishes, birds, mammals, the timid and the bold, all display this characteristic during the reproductive viscus.

> "Omne adeo genus in terris hominumque ferarumque

> Et genus æquoreum, pecudes, pictæ volucres

> In furias ignemque ruunt. Amor omnibus idem."

> VIRGIL: *Georg.*, 242.

The difference between the animal at the season of reproduction and at the time when the instinct is in abeyance, constitutes one of the most striking illustrations of the power of a distant organ over the cerebral functions. In the vertebrata it is not more obvious than in the invertebrata, but it is more curious as a phrenological fact. It cannot be supposed that, during the manifestation of this and connected periodic instincts there is an increase or diminution of the *phrenological* organ in the cerebrum in proportion as the organs of reproduction are active or inactive; to what then must we attribute the change? No other explanation includes all the facts, than the hypothesis that there is an appropriate molecular organization of the cerebrum, which co-ordinates to the intended end, to be put into action by an appropriate stimulus. The latter being applied the former becomes active; just as a bar of iron continues magnetic so long as a magnetic current passes through it. That the stimulus is requisite to the co-ordination, is proved by the results which follow removal of the organs necessary to reproduction. The unmanly expression and manner of the effeminate male is due to the *want* of the stimulus; on the other hand, in viragos the transformation from the feminine to the masculine temper and manner seems due to the shrinking of the ovaria, and probably to the *change* in the nature of the stimulus which they give to the system consequent on that shrinking. That this pathological condition occurs in the ovaria of the female *gallinaceæ*, when they assume the feathers of the cock-bird, has been shown by dissections.

*Vanity* in men and women as to personal appearance, is often seen to be developed as a symptom of insanity. The *Adonis* of the lunatic asylum cultivates the natural ornaments of his sex with sedulous care. Hence the attention devoted to the whiskers, moustache, beard, and hair. A similar characteristic in the female is well-known to popular writers.

> "For never did this maid—whate'er
> The ambition of the hour—forget
> Her sex's pride in being fair;
> Nor that adornment, tasteful, rare,
> Which makes the mighty magnet, set
> In woman's form, more mighty yet."

Perverted, it constitutes, indeed, part of the popular idea of insanity in the female. It is, we need hardly say, always bizarre, absurd, and eccentric in its effects. Who, accustomed to the insane of the gentler sex, has not witnessed over and over again, manifestations of this foible in strangely decorated bonnets, odd caps, curiously quaint ornaments, and the like? The *manufacture* of these ornaments out of the most unpromising materials, is a not less curious development of (as we think) an instinct in insanity. It is in the *female* that the instinct for making up materials is *most* manifested. This is seen even in lower animals. That the preference for ornaments and for feminine work belongs not unfrequently to this class of instincts, is known by its absence in the virago; on the other hand, the ornaments of the male birds of almost every family, and of the males of many animals, far exceed those of the female, and are demonstrably dependent upon the development of the testes.

We ought not to omit one effect of these distant and singular sympathies, namely, the remarkable loss of appetite for food when the amatory impulse is urgent. It is observed in lower animals as well as in man. Stags, and others of the class, are scarcely ever seen to eat during the whole rutting season. "It is a physical observation," says Fielding, in 'Joseph Andrews,' "that love, like other sweet things, is no whetter of the stomach." This anorexia is sometimes developed in a remarkable degree in hysterical girls; Ann Moore, the "fasting woman" of Tutbury, was one of numerous examples that might be mentioned. It is not improbable that various morbid appetites may be only perversions of this instinct for food, induced by ovarian influence, as when the females of lower animals—sows, cats, &c.,—eat their offspring.

*Monomaniacal cunning* in the human female is another instinct morbidly developed by ovarian sympathies. The same influence which makes the male of lower animals quarrelsome during the season of procreation, makes the female cunning and cautious. The skill displayed in the choice of a secret place for the eggs or young, and the finesse practised to lure or scare away the destroyer, are wonderful displays of instinct. The lioness may be mentioned as one illustration of many, for, ferocious as she is, she places no reliance upon her strength to defend her young; but when she fears that the retreat where she has hid them may be discovered by her footprints, she effaces them with her tail. *Klepto-mania*, or the impulse to steal (acquire cunningly), appears to be related to the monomaniacal *cunning* we have just mentioned. Like the latter, the former is generally observed in females, and most frequently during a period of ovarian or uterine activity. These two will occur, indeed, in the same patient, and then the criminal results of the combination are very remarkable; but cunning is more frequent in the hysterical, theft in the pregnant or parturient woman. Both are very compatible with that *modesty* which is a great charm in the sex, and which being itself a sexual characteristic, may be developed concurrently with others of the class. From time to time these monomaniacal impulses are seen in pubescent lads; some of these have been indeed the most successful travelling *illuminati* of the itinerant mesmerizer or electro-biologist. The curious forms which *cunning* of this kind assumes are very interesting, if in compared with the habits and practices of lower animals. In this, as the other instincts, a lower stratum appears to "crop out" in the insane.

There is another faculty under the control of the reproductive organs, of which we ought to say something,—the *instinct for musical sounds* and *rhythmical cadences* which the action of the ovaria and testes excite in a great variety of animals, as well in the *articulata* as the *vertebrata.* The voice is exclusively developed in the males of some animals; in others it is feeble and unmusical in the female; rich, full-toned and melodious in the opposite sex. The horse and horned cattle, the gallinaceæ, and all singing birds, afford ample illustrations of this connexion. In man the instinct is manifested in the rhythmical and harmonious collocation of words and cadences, from the "woeful ballad made to a mistress' eyebrow" to the highest and grandest poesy. An illustration of this amatory instinctive development of the musical faculty, is related in the biography of Paganini. It was love that first made manifest his magical power on the violin; the history is thus given by himself:—

"I was playing at the court of Lucca to the princess (Napoleon's favourite sister) and another fascinating creature, that must be nameless, who, I flatter myself, felt a penchant for me, and was never absent from my performance. On my own side I had long been her admirer. Our mutual fondness gradually became stronger and stronger; but we were forced to conceal it, and by this means its strength and fervour were greatly enhanced. One day, I promised to surprise her, at the next concert, with a musical joke, which should convey an allusion to our attachment; and I accordingly gave notice at court that I should bring forward a musical novelty, under the title of a *Love Scene.* The whole world was on tiptoe; and, on the evening appointed, I made my appearance, violin in hand. I had previously robbed it of the two middle strings, so that none but the E and G remained, the first string being designed to play the maiden's part, and the lowest the youth's. I began with a species of dialogue, in which I attempted to introduce some events analogous to transient bickerings, and reconciliations between the lovers. Now my strings growled, and then sighed; and anon, lisped, hesitated, joked, and joyed, till at last they sported with merry jubilee. Shortly both souls joined once more in harmony, and the appeased lovers' quarrels led to a *Pas de deux*, which terminated in a brilliant *Coda.* This brilliant fantasia of music was greeted with loud applause. The lady, to whom every scene referred, rewarded me by looks of delight, and full of sweetness; and the princess was charmed into such amiable condescension, that she loaded me with encomiums; asking me whether, since I could produce so much with two strings, it would not be possible to gratify them by playing only on one. I yielded instant assent. The idea tickled my fancy; and, as the emperor's birth-day was at hand, I composed a *sonata* for the G string, which I entitled 'Napoleon,' and played before the court with so much effect, that a cantabile, given by Cimarosa, fell without producing any impression upon the hearers. This is the genuine and original cause of my predilection for the G string."*

*Responsive Sympathies.*—By this term we mean to imply that large group of sympathies which are called into operation by sensorial

* "The Music of Nature; or, *an Attempt to prove that what is passionate and pleasing in the art of Singing, Speaking,* and *Performing upon Musical Instruments* is derived from the Sounds of the Animated World." By Wm. Gardiner. London: 1833. Pp. 222.

stimuli derived from without, in which there is some hidden cerebral mechanism that *responds* to the stimulus; and which, in so responding, puts the whole nervous system into co-ordinate action. The entire group of the instincts, emotions, and passions, when developed by sensorial stimuli, or external impressions, become responsive sympathies; but there is a less obvious, but not less important class, which belongs to conceptions and ideas of memory, and to cerebral changes induced by circumstances of which memory retains no trace.

We have seen how uniformly and constantly there are developments in cerebral organization correspondent to the varied stimuli which the organism can receive under any circumstances within the limits of its powers. Some of these developments are *natural*, and have existed in the species *ab origine;* some are *acquired*, and are only recent; many seem to belong (as we have seen) to a lower grade of development; as if the cerebral organization of some antecedent existence had been *transmitted* from a lower life, to reappear and be awakened into action by external impressions when the higher developments are in abeyance. Many, too, we must add, seem to belong, on the other hand, to a higher stage of existence; so that when an appropriate stimulus falls, as it were, haphazard upon the material development, a strange and transient gleam of mysterious consciousness flashes across the mind—an idea—a notion—a vision,—as of something known aforetime, appearing, for a moment, from the depths of the intellect, yet leaving an indefinite but ineffaceable impression. In this way it is that the thoughts become rife—

> "With airy images and shapes which dwell
> Still unimpaired, though old, in the soul's haunted cell."

Perhaps we have something of this kind in the retention by domesticated animals of certain instincts of their wild state; or in the re-appearance of such instincts when the domesticated animal has again become wild. The dog, for example, retains the instinctive act of turning himself round and round, and scratching his bed-mat or bedding before he goes to rest—a useless movement under the circumstances. So also several varieties of dogs retain the instinct of concealing their food in the ground—a wild instinct. The wild horses of South America are descendants of the domestic breed. In them we see the full development of gregarious instincts, which are hardly apparent in the stock from which they have sprung. So also there appear to be in civilized man traces of those ideas which occupied the mind of his uncivilized ancestor, when "wild in woods the noble savage ran." Sir R. H. Bonnycastle observes, in his work on Canada, "The best specimen of an Indian missionary I am acquainted with in Upper Canada forgot all his instruction, all his acquired feelings and habits, when he witnessed with me the war-dance of heathen and perfectly savage warriors. He had been carefully educated from a boy, was modest, intelligent, and well-bred, . . . . . yet he grinned with delight at this exhibition of untutored nature." Who has not felt the wild excitement which comes over the feelings, when wandering free over hill and mountain in the full enjoyment of health? The external stimulus of a trackless surface, the feeling of freedom from social restraint, the fresh breezes, and all the change from town to country existence, touch chords of enjoyment deeply hidden in the soul, and render us competent to understand how it is that the Indian or back-woodsman flees before advancing civilization. In Huc's Travels in Tartary and Thibet, we find an interesting illustration of the existence of dormant sympathies, transmitted from pre-existent ancestorial modes of life, of this kind.

"Among the Lamas who came to recreate for awhile at Tchogortan we remarked," says M. Huc, "especially a number of Tartar Mongols, who, bringing with them small tents, set them up in the valley along the stream, or upon the sides of the most picturesque hills. There they passed whole days, revelling in the delight of the independent life of the nomades, forgetting for a while the constraint and confinement of the Lamanesque life, in the enjoyment of the free life of the tent. You saw them running and frolicking about the prairie like children, or wrestling and exercising in the other sports which recalled the days and the land of their boyhood. The reaction with many of these men became so strong that even fixity of tent was insupportable, and they would take it down, and set it up again in some other place, three or four times a day; or even they would abandon it altogether, and taking their kitchen utensils and their pails of water, and their provisions on their shoulders, would go, singing and dancing as they went, to boil their tea on the summit of some mountain, from which they would not descend until nightfall."

We might multiply illustrations of this kind to an indefinite extent, and trace the springs of action of nations as well as individuals to sympathies deeply writ in the organism, but latent and dormant until the stimulus is applied. We could point out the connexion between these and great social changes, in periods of revolutionary disturbance. It is then that society is agitated to its lowest depths, and dormant instincts, hitherto pressed down by the weight of law and order, spring forth when that weight is taken off and their appropriate stimuli applied, to the astonishment of a wondering world. This branch of psychology is not, however, our present theme. We have now to consider not social but mental disorder, and to say something of

*The Sympathies as curative Agents in Insanity.*—The proposition we have to state on this point may be readily gathered from the preceding remarks. The treatment of insanity in its entirety consists in the application or removal of the stimuli to the sympathies. Of those which are purely corporeal, we have already indicated the relations; we have, therefore, to treat only of the mental. A right knowledge of the uses of these sympathies constitutes the only sound basis of the *moral* treatment of insanity. Therapeutically, we may consider them in their threefold aspect of the instinctive, the emotional, the intelligential. Now, as to the two former, the rule is this: the manifestation of them in one individual excites them in another. If we would excite anger, we must be angry; if we would excite attack, we must attack; if we would excite pleasant friendliness, we must greet friendlily, and show kindliness in actions. How often do we see the magic effect on the insane of a sunny, kindly smile!—

> "Like moonlight o'er a troubled sea,
> Brightening the storm it cannot calm."

Undoubtedly, a gentle winning demeanour towards the violent and sullen, is of very potent curative influence. One of such a temperament (Byron) knew its influence well:—

> "It is in vain that we would coldly gaze
> On such as smile upon us; the heart must
> Leap kindly back to kindness."

The non-restraint system of treating the maniacally violent is founded wholly on this psychology of the instincts and emotions. Personal restraint in the *sane* excites resistance—how much more in the *insane*, in whom the disposition to attack and resist is morbidly excited. It is very worthy note, however, that when personal restraint is *necessary* (as surely it must be in *some* cases of maniacal violence), that restraint must be applied in accordance with this principle. Is it not questionable, then, whether the use of *persons* for this purpose be so advisable as the use of *things?* We doubt, indeed, whether the *attendant* can himself remain perfectly free from emotion when his corporeal energies are called forth to resist the struggles of a violent maniac, so potent and ever active are the stimuli to these instinctive emotions.

The same principle of applying or removing these stimuli is applicable to other instincts and emotions morbidly developed. In a case of suspecting melancholia, a *really* frank and truthful manner and mode of treatment will alone gain the confidence of the patient. The slightest attempt at deception is not only quickly perceived by the morbidly developed instinct, but it immediately excites that morbid development more fully. When the suicidal impulse is the special characteristic, arguments addressed to the judgment will fail, when the perverted or abolished instinct of love of life may be restored by any means which tend to excite it. Thus it will happen that when the patient urgently desires certain means of self-destruction, the impulse may be checked by suggesting to him the choice of other means; e. g., a razor being desired by the monomaniac to cut his throat, if arsenic be suggested to him as an equally effective means, with an explanation of the anguish it inflicts, he would shudder at the idea of self-destruction, since the contemplation of the pain, and of death by other means than that upon which his "fixed idea" dwells, rouses the dormant instinct of love of life and ease. It is from the same causation that a half successful attempt at suicide sometimes cures the patient, or checks the progress of the disorder. An illustration of this kind is mentioned by Dr. Burrows, in his "Commentaries." It is true that *he* attributes the cure to "fever," but it is obvious that it was only *feverishness* from which the patient suffered; and this must be considered as wholly inadequate to the cure of suicidal insanity.—

"A gentleman, aged forty-five, in a state of melancholia, with a strong propensity to suicide, was walking with his keeper on Battersea Bridge. By a sudden effort, he broke away and jumped into the Thames. It was on a Sunday, and as many boats were passing in the river, assistance was immediately given; but he resisted so much, that it was only by main force he was taken out of the water and conveyed to his residence. Having some distance to go in his wet clothes, he caught a violent cold, followed by rigors and a smart fever. For this, I prescribed suitable remedies; but I took no notice, nor made any inquiry of him respecting his late rash attempt to destroy himself. During the fever, he was quite docile and collected. When it had subsided, I reasoned with him on the subject. He confessed himself horror-struck on the reflection of the act he had committed, and entreated I never would again mention it. In fact, his mind was entirely free from all delusion; and in a fortnight he returned home cured, and has remained well ten years."

Dr. Burrows quotes this case as an illustration of the curative effects of "fever," with the object of supporting a theory of the relations of the cerebral circulation to mental disorder. That insane patients have recovered after attacks of fever is certain, but the order of events is doubtful; it *may be* that the functional changes in the circulation within the cerebrum begin the cure in some, but in others (as the *suicidal* forms), it *may be* that the vis conservatrix, having been roused by the fever into action, has extended its influence to the instinct of love of life, and restored it to orderly impulses.

The case just quoted may serve, as well as another, to illustrate that part of the moral treatment of insanity, which consists in the *removal* of stimuli. In a vast majority of cases of destructive insanity, from impulse, the impulse is developed by the presence of the object to be destroyed (when it is a thing or another than the *self*), and of the

*means* adopted to the destruction desired. These constitute the *stimuli* of the instinct. In Dr. Burrows's case, the flowing river excited the paroxysm; indeed, it is a well-known practical point in the construction of asylums, to avoid a site within reach or view of a river or of water. A case equally illustrative of the general principle, once came under our own notice. A female, aged about 35, delivered three months ago of her third child, consulted us about the "temptations" to which she was exposed. She explained that they came on in an instant, as quick as lightning, and after continuing for a moment would pass off. When nursing her infant she is tempted, in this instantaneous manner, to dash it to the ground, trample on it, and destroy it. She is also tempted to destroy herself, sometimes in one way, sometimes in another. Going through a passage to the house, for example, she is suddenly tempted to dash her head against the wall, or if she see a knife on the table when the child is in her arms, she feels a sudden desire to seize the knife and cut the child's throat. When other instincts are roused, of which the stimuli are less sensational and more intelligential, the connexion between cause and effect is not so obvious, but can nevertheless be traced by a careful analysis. The most remarkable instance of moral insanity we ever met with, was in a young girl, who combined *cunning* with the impulse to suicide, cruelty, and destruction of life. She was of very simple manners; and being modest in her demeanour and language, never gave an indication of the latent devil within her; but when at all under the influence of cerebral excitement, she would snatch at or pinch any one passing her, or kick at those near her—without, however, betraying the slightest change of gesture. By pressing blandishments she would persuade some old and feeble person to go into a corner, or other convenient spot, out of hearing of those around her, and then changing her manner, endeavour to get them to deny their Saviour, on pain of the most terrible vengeance. When she succeeded (as she did occasionally), she would dance about and seem delighted with the idea, which she loudly expressed, that the object of her cruelty would now be sure to go to hell. It was quite unsafe to leave her alone with a child or a feeble patient, as she would immediately most cunningly plot their destruction; and it was believed that she had succeeded in one instance, if not two. She often attempted suicide. It always appeared that *to be left alone* was the great stimulus to her suicidal or homicidal attempts, and that the chain of effects began with the impulse to secrecy and cunning.

The instincts and emotions in relation with love of offspring or of the opposite sex are easily amenable to stimuli. Hence it is that children are such admirable curative agents in an asylum; hence the advantage of balls and concerts. Very often we have noticed in a class of patients, not uncommon in asylums, namely, young females in a state of amentia with dirty habits, and displaying an utter neglect of the person, that the first awakening of the dormant intellect has been due to the presentation of some trifling ornament of the hair or person, or to some other means by which the instinct for personal adornment has been awakened. This has proved the key note to the disordered chords of the imagination, and with the progress of this development from maniacal finery to rational neatness and good taste in dress, other faculties have been *pari passu* evolved.

*The intellectual sympathies* are very various; sometimes they are known from the previous habits and thoughts of the insane; sometimes they are accidentally discovered by the chance application of the appropriate stimulus. Of the latter there is an illustration in Crabbe's tale of Edward Shore.—"A harmless wretch beyond a cure;" he wanders abroad, and "that gentle maid whom once the youth had loved" pities him.

> "Kindly she chides his boyish flights, while he
> Will, for a moment, fixed and pensive be;
> And as she trembling speaks, his lively eyes
> Explore her looks, he listens to her sighs;
> Charmed by her voice, th' harmonious sounds invade
> His clouded mind, and for a time persuade;
> Like a pleased infant, who has newly caught
> From the maternal glance a gleam of thought;
> He stands enrapt, the half-known voice to hear,
> And starts, half conscious, at the falling tear."

In developing a large class of this group of sympathies, the *imitative* instinct may be brought into beneficial operation. If a patient be placed amongst others actively engaged in various occupations, the desire will sooner or later arise to imitate them, and that employment will probably be selected which in days of mental health and vigour has been a favourite or habitual pursuit. Thus it is that industrial employments have so happy an influence on the insane; thus also we comprehend how music may arouse some long-forgotten thoughts and wake up mental life again. Perhaps country life and rural scenes exercise a secret beneficial influence rather than a visible effect, by their revival of those sympathies with nature which are rarely absent from the most uncongenial soul, but which are often the root of many noble and pleasant aspirations, and deeply felt by those predisposed to cerebral disease.

> "There is a pleasure in the pathless woods,
> There is a rapture on the lonely shore,
> There is society where none intrudes
> By the deep sea, and music in its roar.

The character of the emotions which these impressions excite, is proof of their innate instinctive origin. It was in his "interviews" with nature that Byron experienced the pleasure he so well describes; he stole forth to them—

> "To mingle with the Universe, and feel
> What I can ne'er express, yet cannot all conceal."

If, while these soothing, gentle stimuli be presented to the mind, it be drawn at once from those of the cares of business, and the anxieties of worldly affairs, moral treatment is of essential advantage, and under these circumstances the asylum is emphatically and truly a retreat.

---

